# Global biomarkers of oxidative stress and fractures: a matched case-control study

**DOI:** 10.3389/fendo.2023.1179521

**Published:** 2023-06-23

**Authors:** Shuman Yang, Lijie Feng, Lisa M. Lix, William D. Leslie, Dingjie Guo, Xianbao Shi, Baoming Yuan

**Affiliations:** ^1^ Department of Epidemiology and Biostatistics, School of Public Health, Jilin University, Changchun, Jilin, China; ^2^ Department of Orthopedics, The First Affiliated Hospital of Jinzhou Medical University, Jinzhou, Liaoning, China; ^3^ Department of Community Health Sciences, University of Manitoba, Winnipeg, MB, Canada; ^4^ Department of Internal Medicine, University of Manitoba, Winnipeg, MB, Canada; ^5^ Department of Pharmacy, The First Affiliated Hospital of Jinzhou Medical University, Jinzhou, Liaoning, China; ^6^ Department of Orthopedics, The Second Hospital of Jilin University, Changchun, Jilin, China

**Keywords:** oxidative stress, fluorescent oxidation products, osteoporosis, fracture, Chinese, hip fractures, non-hip fractures, case-control study

## Abstract

**Background:**

Evidence for a relationship between oxidative stress and osteoporotic fractures in humans is limited. Fluorescent oxidation products (FlOPs, excitation/emission wavelengths 320/420nm denoted FlOP_320; 360/420nm [FlOP_360]; and 400/475nm [FlOP_400]) are global biomarkers of oxidative stress, and reflect oxidative damage to proteins, phospholipids, and nucleic acids. We investigated the association between FlOPs and a recent osteoporotic fracture.

**Methods:**

We conducted a case-control study in a Chinese population aged 50 years or older. A recent osteoporotic fracture in the cases was confirmed by x-ray. Cases were matched with community-based non-fracture controls (1:2 ratio) for age (± 4 years) and sex. In addition, we conducted a sensitivity unmatched case-control study which included all fracture cases and all eligible non-fracture controls prior to matching. Plasma FlOPs were measured with a fluorescent microplate reader. We used unconditional logistic regression to analyze the association between FlOPs (per 1-SD increase in logarithmic scale) and fracture; odds ratios (OR) and 95% confidence intervals (95% CI) were reported.

**Results:**

Forty-four cases and 88 matched controls (mean age: 68.2 years) were included. After covariate adjustment (i.e., body mass index, physical activity, and smoking), higher FlOP_360 (OR = 1.85; 95% CI = 1.03 – 3.34) and FlOP_400 (OR = 13.29; 95% CI = 3.48 – 50.69) levels, but not FlOP_320 (OR = 0.56; 95% CI = 0.27 – 1.15), were associated with increased fracture risk. Subgroup analyses by fracture site and unmatched case-control study found comparable associations of FlOP_360 and FlOP_400 with hip and non-hip fractures.

**Conclusions:**

Higher FlOP_360 and FlOP_400 levels were associated with increased risk of fracture, and this association was comparable for hip and non-hip fractures. Prospective studies are warranted to confirm this finding.

## 1 Introduction

Oxidative stress (OS) is defined as a state of imbalance in which reactive oxygen species (ROS) cannot be reduced by endogenous antioxidant defense mechanisms (1). OS is a risk factor for many chronic diseases such as asthma, coronary heart disease, and cancer (2–4).

Studies suggest that OS disturbs the balance of osteoclasts and osteoblasts, and increases the risk of osteoporosis *via* the Wnt/β-catenin and RANKL/RANK/OPG pathways (5, 6). However, human evidence for a relationship between OS and osteoporotic fractures is limited. Most human epidemiologic studies suggest a negative association between uric acid (a potential antioxidant) and fracture risk (7). The Norwegian Epidemiologic Osteoporosis Studies (NOREPOS) found that lower serum alpha-tocopherol levels were associated with increased hip fracture risk in older Norwegians (8).

Traditional biomarkers of OS include malondialdehyde (MDA), 8-hydroxy-2 deoxyguanosine (8-OHdG), pentosidine (PTD) and nitrotyrosine (NT), which reflect oxidative damages from lipids, DNA, carbohydrates and protein, respectively. As compared to traditional OS biomarkers, plasma fluorescent oxidation products (FlOPs, excitation/emission wavelengths 320/420nm named as FlOP_320; 360/420nm named as FlOP_360; and 400/475nm named as FlOP_400) are global biomarkers of oxidative stress, and reflect oxidative damage to proteins, phospholipids, and nucleic acids combined (9). A nested case-control study from the United States demonstrated that higher FlOP_320 levels were associated with increased risk of hip fracture among postmenopausal women (10). However, it is unclear whether FlOPs are associated with non-hip fractures or if the FlOP-fracture relationship exists in other populations. Chinese population has different oxidative stress levels (11, 12), fracture incidence rates (13, 14), and distributions of fracture related factors (i.e., lifestyle factors) (15) compared with the US population.

Osteoporotic fractures are a major public issue in China. The incidence rates of major osteoporotic fractures and hip fracture in China were 120 and 50 per 100,000 person-years in males and 213 and 43 per 100,000 person-years in females, respectively; with individuals aged 55 years or above most heavily impacted (16). The costs of osteoporotic fractures are projected to be 25 billion by 2050 (17). The one-year mortality rate following a hip fracture is approximately 14% (18). Therefore, we investigated the association between FlOPs and osteoporotic fractures, including both hip and non-hip fractures, in a Chinese population.

## 2 Materials and methods

### 2.1 Study setting and participants

**Figure 1:**
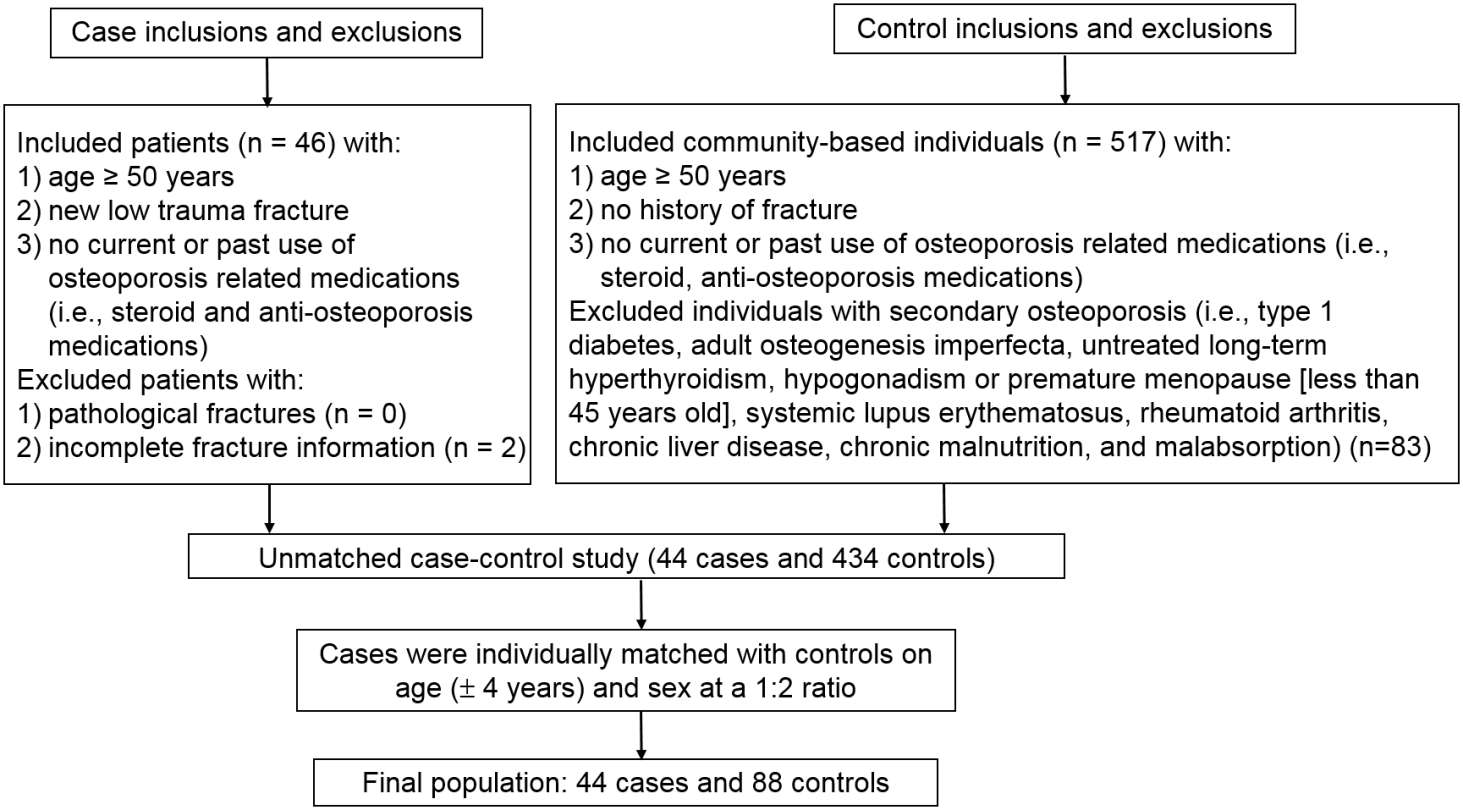
Study flowchart.

Cases aged 50 years or older with a new hospitalized low-trauma fracture were identified from the Department of Orthopedics, Second Hospital of Jilin University, Changchun, Jilin in 2020 (**Figure 1**). All fractures including hip, forearm and humerus fractures were confirmed by x-ray. Causes of fractures (falls, low-trauma sports injury and others) were also coded. We excluded cases with pathological fractures or incomplete fracture information (i.e., date or site of fracture). Fracture-free controls aged 50 years or older were selected from a community-based generally healthy population in Changchun, Jilin in 2020. We excluded controls with conditions associated with secondary osteoporosis (i.e., type 1 diabetes, adult osteogenesis imperfecta, untreated long-term hyperthyroidism, hypogonadism or premature menopause before age 45 years, systemic lupus erythematosus, rheumatoid arthritis, chronic liver disease, chronic malnutrition, or malabsorption). Cases and controls were restricted to those with no current or past use of osteoporosis-related medications (i.e., steroid and anti-osteoporosis medications). Cases were individually matched with controls by age (± 4 years) and sex at a 1:2 ratio. In addition to the primary matched case-control study, we conducted a sensitivity unmatched case-control study which included all fracture cases and all eligible non-fracture controls prior to matching. All participants provided written informed consent. This project was approved by the institutional review boards (IRBs) of the School of Public Health, Jilin University (Project #: 2018-12-06) and the Second Hospital of Jilin University (Project #: 2018-10-10). Based on a pilot study of 4 fracture cases and 8 controls, and their corresponding FlOP_400 levels (73 ± 3 FI/ml in cases and 60 ± 10 FI/ml in controls), to achieve study power > 0.80 with α = 0.05, we estimated a minimum sample size for cases and controls of 12 and 24, respectively. FlOP_400 were used in sample size estimation because they showed a larger difference than other FlOPs between the cases and controls.

### 2.2 Blood collection

After fasting for a minimum of 8 hours (except for water), we collected blood samples with 5 ml heparin anticoagulant tubes (Catalog no: 367884, BD, New Jersey). Blood collection for cases was completed prior to fracture treatment, and within one to two days following the hospitalization for fracture. All blood sample were stored on ice and transported to the laboratory of Jilin University within 8 hours. These blood samples were processed and divided into aliquots and stored in a -80°C freezer until assay.

### 2.3 Measurement of FlOPs

The measurement method for FlOPs in plasma has been described in previous studies (10, 11). Briefly, plasma samples were mixed with ethanol: ether (3:1, v/v) at 1:20 (v/v) ratio. We centrifuged mixtures at 3000 rpm at 4°C for 10 minutes to obtain supernatant and the supernatant was measured on 96-well Corning plates (Product number: 3925, Corning^®^, New York, USA) at three excitation/emission wavelengths (see above) with a fluorescent microplate reader (Cytation 3 Cell Imaging Multi-Mode reader from Bio-Tek, Vermont, USA). FlOP_320 represent the interaction of lipid oxidative products with DNA and metals, FlOP_360 are generated from oxidized phospholipids or from lipid oxidation products reacting with proteins, DNA, and carbohydrates in the presence of phospholipids, and FlOP_400 reflects the interaction between MDA, proteins, and phospholipids (19). The intra- and inter-assay coefficients of variation for FlOP measurements were < 1.7% and 3.3%, respectively.

### 2.4 Ascertainment of covariates

The covariates included demographics (sex and age), lifestyle factors (i.e., smoking status, milk intake frequency, calcium supplement intake, and physical activity), postmenopausal status in females, disease history (i.e., coronary heart disease, type 2 diabetes, and stroke), height loss more than 3 cm after age 40 years, falls from standing height or less within the last 12 months, family history (i.e., osteoporosis and fracture) and body mass index (BMI). These covariates were selected because they are established risk factors for fracture and/or osteoporosis (20, 21). The disease histories were obtained from electronic medical records in cases and face-to-face interview in controls; all other information was collected through a face-to-face interview. Physical activity, expressed as metabolic equivalent hours per week (MET-hours/week), was calculated from frequency and duration of light, moderate, and vigorous physical activities (22). For cases, body weight and height were self-reported, whereas for controls, body weight and height were directly measured. BMI was calculated as body weight (kg) divided by body height squared (m^2^). Data on c-reactive protein, white blood cell count and neutrophil count were also extracted from electronic medical records for cases.

### 2.5 Statistical analysis

Baseline characteristics for cases and controls were descriptively analyzed for matched and unmatched case-control studies. We also descriptively analyzed the skeletal site, biochemical test results and causes for cases. Pearson correlations of FlOPs with c-reactive protein, white blood cell count and neutrophil count in cases were tested for statistical significance. We further examined the associations of the baseline characteristics with FlOP_320, FlOP_360 and FlOP_400 in multivariable linear regression models; these characteristics included age, sex, BMI, physical activity, smoking, milk intake >1 time/week, calcium supplement, history of coronary heart disease, history of type 2 diabetes, history of stroke, height loss >3 cm, falls, family history of osteoporosis and family history of fractures. Results are reported as regression coefficients with *P* values.

As suggested by a previous study (23), we used unconditional logistic regression models to test the association between FlOPs and fractures and reported odds ratios (ORs) and 95% confidence intervals (CI). Model 1 was adjusted for BMI, physical activity, milk intake >1 time/week and falls; these factors showed associations with cases at alpha=0.1 in bivariate analyses. Model 2 was adjusted for age, sex, BMI, physical activity, smoking, milk intake, calcium supplement, history of coronary heart disease, history of type 2 diabetes, history of stroke, height loss >3 cm, falls, family history of osteoporosis and family history of fractures. Using the above two models will allow us to test the stability of results after adjusting for different covariates. We treated FlOPs as continuous variables (per 1-SD increase in logarithmic scale). Subgroup analyses by fracture type (hip fracture vs. non-hip fracture) were also conducted. In these subgroup analyses, we used all controls to increase the study power and adjusted for all covariates as above. Lastly, we tested for effect modification of physical activity, milk intake >1 time/week and falls with FlOPs by including two-way interaction terms (physical activity*FlOPs, milk intake >1 time/week*FlOPs and falls*FlOPs). Again, these analyses were performed in matched and unmatched case-control studies. All analyses were performed with the SPSS software (version: 24.0; SPSS, Chicago, IL).

## 3 Results

Forty-four fracture cases and 88 non-fracture controls were included in the matched case-control study; 44 fracture cases and 434 non-fracture controls were included in the unmatched case-control study (**Figure 1**). Skeletal site, biochemical test results and causes of fracture cases are shown in **Supplemental Table 1**. Among the 44 cases, 23 (52.3%) were hip fracture patients. Falls, low-trauma sports injury and other reasons accounted for 38.6%, 38.6% and 22.7% of fractures. FlOPs were not significantly correlated with c-reactive protein, white blood cell count and neutrophil count in cases (**Supplemental Table 2**). In the matched case-control study, cases had lower physical activity levels and frequency of milk intake, and higher percentage of falls than controls (**Table 1**). FlOP_400 levels were higher in cases than in controls. The numbers of postmenopausal females in female cases and controls were 33 (100%) and 63 (95.5%), respectively (*P* for difference = 0.292). Other characteristics (age, sex, BMI, smoking, calcium supplement use, history of coronary heart disease, history of type 2 diabetes, history of stroke, height loss >3 cm, family history of osteoporosis, and family history of fractures) were comparable between cases and controls. In the unmatched case-control study, cases tended to be older, female and had lower BMI, physical activity, frequency of milk intake, and higher frequency of falls and history of stroke as compared to controls (**Supplemental Table 3**). FlOP_360 and FlOP_400 levels were higher in cases than in controls.

**Table 1:** Baseline characteristics of individuals by fracture status in the matched case-control study.

Characteristic	Fracture (*N* = 44)	Non-fracture (*N* = 88)	P
Age (years)^a^	68.2 (10.0)	68.2 (9.8)	0.995
Female (n, %)	33 (75)	66 (75)	>0.999
Body mass index (kg/m^2^) ^a^	23.5 (3.6)	24.9 (3.9)	0.056
Physical activity (MET‐hours/week)^b^	19.7 (4.4, 26.3)	35.0 (30.6, 48.1)	<0.001
Smoking (n, %)	7 (15.9)	6 (6.8)	0.124^c^
Milk intake >1 time/week (n, %)	31 (70.5)	77 (87.5)	0.017
Calcium supplement (n, %)	17 (38.6)	28 (31.8)	0.436
History of coronary heart disease (n, %)	6 (13.6)	18 (20.5)	0.338^c^
History of type 2 diabetes (n, %)	9 (20.5)	21 (23.9)	0.660
History of stroke (n, %)	5 (11.4)	4 (4.5)	0.159^c^
Height loss >3 cm (n, %)	22 (50.0)	44 (50.0)	>0.999
Falls (n, %)	17 (38.6)	14 (15.9)	0.004
Family history of osteoporosis (n, %)	1 (2.3)	7 (8.0)	0.268^c^
Family history of fractures (n, %)	5 (11.4)	7 (8.0)	0.533^c^
FlOP_320 (FI/ml) ^b^	127 (116, 158)	141 (128, 160)	0.066
FlOP_360 (FI/ml) ^b^	121 (110, 139)	113 (103, 123)	0.083
FlOP_400 (FI/ml) ^b^	39.5 (36.0, 45.0)	33.5 (30.1, 36.3)	<0.001

Unless otherwise specified, ^a^variables with normal distribution are presented as means (standard deviations); ^b^variables with skewed distribution are shown as medians (interquartile ranges). ^c^Fisher’s exact test was used.

MET, metabolic equivalent task.

In the multivariable analysis, FlOP_320 and FlOP_360 was not associated with any baseline characteristics (**Supplemental Table 4**). Age was positively associated with FlOP_400 (*P* = 0.014). Physical activity was negatively associated with FlOP_400 (*P* = 0.006).

In the matched case-control study, after adjusting for model 2 covariates, higher FlOP_360 (OR = 1.85; 95% confidence interval [CI] = 1.03 – 3.34) and FlOP_400 (OR = 13.29; 95% CI = 3.48 – 50.69) levels, but not FlOP_320 (OR = 0.56; 95% CI = 0.27 – 1.15), were significantly associated with higher risk of osteoporotic fracture (**Table 2**). Similar results were noted when we adjusted for model 1 covariates. Physical activity, milk intake >1 time/week and falls did not modify the associations between FlOPs and fractures (all *P* for interaction > 0.05). Subgroup analyses by fracture site showed similar associations of FlOP_360 and FlOP_400 with hip fracture and non-hip fracture (**Table 3**).

**Table 2:** Associations between fluorescent oxidation products (FlOPs; per 1-SD increase in logarithmic scale) and fracture in the matched case-control study: Odds ratios (OR) and 95% confidence intervals (CI) from unconditional multivariable logistic regression models*.

FlOPs	Model 1		Model 2	
	OR (95%CI)	P	OR (95%CI)	P
FlOP_320	0.72 (0.37, 1.42)	0.349	0.56 (0.27, 1.15)	0.115
FlOP_360	1.66 (0.98, 2.81)	0.061	1.85 (1.03, 3.34)	0.040
FlOP_400	4.95 (2.18, 11.27)	<0.001	13.29 (3.48, 50.69)	<0.001

FI, fluorescent intensity. *Model 1 was adjusted for body mass index, physical activity, milk intake >1 time/week and falls; model 2 was adjusted for age, sex, body mass index, physical activity, smoking, milk intake, calcium supplement, history of coronary heart disease, history of type 2 diabetes, history of stroke, height loss >3 cm, falls, family history of osteoporosis, and family history of fractures.

**Table 3:** Association between fluorescent oxidation products (FlOPs; per 1-SD increase in logarithmic scale) and recent low-trauma fracture by fracture site in the matched case-control study: Odds ratios (OR) and 95% confidence interval (95%CI) from unconditional multivariable logistic regression models*.

FlOPs	FlOPs in Cases (Fl/ml) Median (Interquartile Range)	FlOPs in Controls (Fl/ml) Median (Interquartile Range)	OR (95%CI)^*^	P^*^
Non-hip fracture (number of cases=21; number of controls= 88)				
FlOP_320	142 (118, 162)	141 (127, 160)	0.67 (0.28, 1.61)	0.369
FlOP_360	122 (112, 139)	113 (103, 123)	1.78 (0.97, 3.25)	0.062
FlOP_400	39.0 (36.0, 45.0)	33.5 (30.1, 36.3)	21.07 (3.38, 131.31)	0.001
Hip fracture (number of cases=23; number of controls= 88)				
FlOP_320	123 (113, 137)	141 (128, 160)	0.42 (0.10, 1.87)	0.256
FlOP_360	119 (109, 134)	113 (103, 123)	1.71 (0.88, 3.33)	0.116
FlOP_400	41.0 (36.0, 45.0)	33.5 (30.1, 36.3)	25.78 (2.68, 248.24)	0.005

FI, fluorescent intensity. *Models were adjusted for age, sex, body mass index, physical activity, smoking, milk intake, calcium supplement, history of coronary heart disease, history of type 2 diabetes, history of stroke, height loss >3 cm, falls, family history of osteoporosis, and family history of fractures.

In the unmatched case-control study, we found positive associations of FlOP_360 and FlOP_400 with fracture (**Supplemental Table 5**). Analysis stratified by hip and non-hip fracture site showed similar significant associations (**Supplemental Table 6**).

## 4 Discussion

In this Chinese population, we found that higher levels of FlOP_360 and FlOP_400 had a positive association with recent low-trauma fractures in the matched and unmatched case-control studies. The association of FlOP_360 and FlOP_400 with hip fracture was comparable with its association with non-hip fracture in the matched and unmatched case-control studies. This suggests that the impact of OS on bone and fracture risk is not site-dependent. Future studies are warranted to confirm these findings.

To the best of our knowledge, this is the first study examining the association of FlOPs with hip and non-hip fractures. This extends our understanding about the effect of oxidative stress on osteoporotic fractures. In our study, fractures were associated with FlOP_360 and FlOP_400, but not with FlOP_320. This contrasts with a previous study, in which there was a positive correlation between FlOP_320 and hip fracture in postmenopausal women (10). The reasons for this are unclear. This may be related to differences in the study populations.

Although both FlOP_360 and FlOP_400 were independently associated with fracture risk, FlOP_400 tended to have a stronger relationship with fracture risk than FlOP_360 (adjusted ORs: 13.29 vs. 1.85 in the matched case-control study; 3.83 vs. 1.73 in the unmatched case-control study). Again, the underlying reasons for this finding remain unclear. However, our study suggests that FlOP_400 may be a better OS marker than FlOP_360 for assessing fracture risk. Certainly, this warrants further confirmation.

In our study, FlOPs were not significantly correlated with c-reactive protein, white blood cell count or neutrophil counts. Similar findings have been found in previous studies (3, 24). This partly supports that FlOPs are specific OS markers, and not markers of inflammation.

There are several potential mechanisms for the positive association of FlOP_360 and FlOP_400 and osteoporotic fractures. FlOP_360 are generated from oxidized phospholipids or from lipid oxidation products reacting with proteins, DNA, and carbohydrates in the presence of phospholipids, and FlOP_400 mainly reflects oxidative damage from the interaction of MDA, proteins and phospholipids (25), where MDA is one of many lipid peroxidation products (26). Increased protein and phospholipid fatty acid oxidation diminishes pro-osteogenic Wnt signaling in the skeleton, which attenuates osteoblast differentiation and promotes osteoblastic cell apoptosis (27, 28). In addition, LDL oxidation products promote loss of bone by directing progenitor marrow stromal cells to undergo adipocyte differentiation (29), and 8-Isoprostaglandin E2 enhanced receptor-activated NF-kappa B ligand (RANKL)-dependent osteoclastic activity of marrow hematopoietic precursors *via* the cAMP-dependent protein kinase pathway (30). *In vitro* cell experiments demonstrate that protein phosphatase 2A (PP2A), a major protein phosphatase in mammalian cells, mediates OS-induced apoptosis in osteoblasts by inactivation of AKT/mTOR pathway, and lipid peroxidation products (4-HNE) may induce OS, inflammatory reactions, and apoptosis in osteoblasts (31).

We found that age was positively associated with FlOP_400. This has also been shown in a previous study (12). In addition, higher levels of physical activity were associated with lower levels of FlOP_400. This contrasts with a previous study (12), in which there was no significant association between FlOP_400 and physical activity. This may also be attributed to the different study populations as above.

Strength and limitations of this study are acknowledged. The fracture cases in this study were recent and objectively confirmed on x-ray. A limitation of this study was the small number of fracture cases. In addition, all cases were recent hospitalized fracture patients, which may lead to increased oxidative stress compared with non-fracture controls from the community. However, Pesic et. al., suggested oxidative stress biomarkers, such as lipid peroxidation, nitrite, superoxide anion radical, and hydrogen peroxide, did not change significantly following a hip fracture (32). Due to the inconvenience of fracture patients, body weight and height in cases were self-reported. However, the finding that cases had lower BMI than controls is consistent with other studies (33, 34). Although participants with steroids were excluded from the study, information on other medications (i.e., statins) was not available. Subclinical conditions, vitamin D intake and bone mineral density data were not available. Lastly, our study did not include a group of hospitalized patients without fractures to complement results from generally healthy controls from the community.

## 5 Conclusion

We demonstrated that FlOP_360 and FlOP_400 were independent risk factors for recent osteoporotic fractures in matched and unmatched case-control studies, and this association was not fracture site dependent. This confirms the harmful effects of OS on bone health. FlOP_360 and FlOP_400 as a global biomarker of oxidative stress may help to refine fracture assessment if our findings are confirmed in prospective studies.

## Data availability statement

The datasets generated and/or analysed during the current study are not publicly available due to ethical reasons but are available from the corresponding author on reasonable request.

## Ethics statement

The studies involving human participants were reviewed and approved by the institutional review boards (IRBs) of the School of Public Health, Jilin University (Project #: 2018-12-06) and the Second Hospital of Jilin University (Project #: 2018-10-10). The patients/participants provided their written informed consent to participate in this study.

## Author contributions

Conception, design, and analysis (SY, LF and BY); interpretation of data (all authors); drafting the article (SY and LF); funding acquisition (SY and BY); critically revising the article for important intellectual content (all authors); final approval of the version to be published (all authors); and agreement to be accountable for all aspects of the work (all authors).
