# Supplementary material for: Global biomarkers of oxidative stress and fractures: a matched case-control study
Source: Front Endocrinol (Lausanne). 2023 Jun 23;14:1179521. doi: 10.3389/fendo.2023.1179521 (PMC10338181; doi:10.3389/fendo.2023.1179521)
Supplement: Supplementary file 1 [file Table_1.docx]

Supplementary Material

**Global Biomarkers of Oxidative Stress and Fractures: A Matched Case-Control Study**

Shuman Yang^a^, Lijie Feng^a^, Lisa M. Lix^b^, William D. Leslie^c^, Dingjie Guo^a^, Xianbao Shi^d^, Baoming Yuan^e,*^

^a^Department of Epidemiology and Biostatistics, School of Public Health, Jilin University, Changchun, Jilin, China; ^b^Department of Community Health Sciences, University of Manitoba, Winnipeg, Manitoba, Canada; ^c^Department of Internal Medicine, University of Manitoba, Winnipeg, Manitoba, Canada; ^d^Department of Pharmacy, The First Affiliated Hospital of Jinzhou Medical University, Jinzhou, Liaoning, China; ^e^Department of Orthopedics, The Second Hospital of Jilin University, Changchun, Jilin, China.

****Address for Correspondence***

Dr. Baoming Yuan

218 Ziqiang Street,

Department of Orthopaedics, The Second Hospital of Jilin University

Changchun, Jilin

China 130000

Phone: +86 13844809599

Fax: +86 043181136747

Email: yuanbm@jlu.edu.cn

**Supplemental Table 1. Skeletal site, biochemical test results and causes of cases**

| **Variable** | **Value** |
| --- | --- |
| **Skeletal site** |  |
| Hip (n, %) | 23 (52.3) |
| Forearm (n, %) | 11 (25) |
| Humerus (n, %) | 10 (22.7) |
| **Biochemical test** |  |
| C-reactive protein (mg/L) | 5.7 (2.2, 40.6) |
| White blood cell count (10^9^/L) | 9.3 (2.6) |
| Neutrophil count (10^9^/L) | 7.3 (2.7) |
| **Causes** |  |
| Falls (n, %) | 17 (38.6) |
| Low-trauma sports injury (n, %) | 17 (38.6) |
| Others (n, %) | 10 (22.7) |

C-reactive protein data were only available in 28 cases, and are shown as median (inter-quartile range). Unless otherwise specified, data are shown as means (standard deviations).

**Supplemental Table 2. Pearson correlations of FlOPs with c-reactive protein, white blood cell count and neutrophil count in cases**

| **FlOPs** | **C-reactive Protein** | **White Blood Cell Count** | **Neutrophil Count** |
| --- | --- | --- | --- |
| FlOP_320 | 0.03 (0.896) | 0.22 (0.177) | 0.16 (0.347) |
| FlOP_360 | 0.09 (0.660) | 0.08 (0.648) | 0.06 (0.729) |
| FlOP_400 | 0.14 (0.485) | 0.14 (0.415) | 0.08 (0.612) |

Values are shown as r (*P*). C-reactive protein data were only available in 28 cases.

**Supplemental Table 3. Baseline characteristics of individuals by fracture status in the unmatched case-control study**

| **Characteristic** | **Fracture**  **(*N* = 44)** | **Unmatched Controls**  **(*N* = 434)** | ***P***^c^ |
| --- | --- | --- | --- |
| Age (years)^a^ | 68.2 (10.0) | 64.7 (10.8) | 0.041 |
| Female (n, %) | 33 (75) | 251 (58) | 0.003 |
| Body mass index (kg/m^2^) ^a^ | 23.5 (3.6) | 24.9 (3.6) | 0.021 |
| Physical activity (MET‐hours/week)^b^ | 19.7 (4.4, 26.3) | 32.6 (2.1, 44.1) | <0.001 |
| Smoking (n, %) | 7 (15.9) | 69 (15.9) | >0.999^d^ |
| Milk intake >1 time/week (n, %) | 31 (70.5) | 373 (85.9) | 0.007 |
| Calcium supplement (n, %) | 17 (38.6) | 125 (28.8) | 0.174 |
| History of coronary heart disease (n, %) | 6 (13.6) | 75 (17.3) | 0.675^d^ |
| History of type 2 diabetes (n, %) | 9 (20.5) | 124 (28.6) | 0.252 |
| History of stroke (n, %) | 5 (11.4) | 9 (2.1) | 0.006^d^ |
| Height loss >3 cm (n, %) | 22 (50.0) | 191 (44.0) | 0.446 |
| Falls (n, %) | 17 (38.6) | 64 (14.8) | < 0.001 |
| Family history of osteoporosis (n, %) | 1 (2.3) | 32 (7.4) | 0.963^d^ |
| Family history of fractures (n, %) | 5 (11.4) | 22 (5.1) | 0.091^d^ |
| FlOP_320 (FI/ml) ^b^  FlOP_360 (FI/ml) ^b^  FlOP_400 (FI/ml) ^b^ | 127 (116, 158)  121 (110, 139)  39.5 (36.0, 45.0) | 143 (129, 161)  113 (103, 125)  32.9 (30.2, 36.5) | 0.027  0.013  <0.001 |

Unless otherwise specified, ^a^variables with normal distribution are presented as means (standard deviations); ^b^variables with skewed distribution are shown as medians (interquartile ranges). ^c^*P* were significance values of unmatched controls vs. fracture cases shown in Table 1. ^d^*Fisher’s* exact test was used.

Abbreviation: MET = metabolic equivalent task.

**Supplemental Table 4. Association between individual characteristics and fluorescent oxidation products (FlOPs) in the matched case-control study: Results from multivariable linear regression models^*^**

| **Variable** | **Unit of Change** | **FlOP_320** | | **FlOP_360** | | **FlOP_400** | |
| --- | --- | --- | --- | --- | --- | --- | --- |
|  | **or Comparison** | **β** | ***P*** | **β** | ***P*** | **β** | ***P*** |
| Age (years) | 10 | -10.19 | 0.404 | 4.04 | 0.453 | 1.57 | 0.027 |
| Female | Yes vs. No | 21.13 | 0.444 | 2.52 | 0.836 | -2.08 | 0.192 |
| Body mass index (kg/m^2^) | 4 | 3.67 | 0.752 | 0.935 | 0.856 | 0.29 | 0.665 |
| Physical activity (MET‐hours/week) | 1 | -0.06 | 0.915 | -0.16 | 0.478 | -0.08 | 0.012 |
| Smoking | Yes vs. No | -4.58 | 0.906 | 2.69 | 0.875 | 2.02 | 0.368 |
| Milk intake >1 time/week | Yes vs. No | 26.50 | 0.377 | -1.89 | 0.886 | -2.90 | 0.094 |
| Calcium supplement intake | Yes vs. No | -1.163 | 0.960 | -4.32 | 0.672 | 0.53 | 0.693 |
| History of coronary heart disease | Yes vs. No | -10.41 | 0.727 | 23.53 | 0.076 | 0.53 | 0.756 |
| History of type 2 diabetes | Yes vs. No | 33.62 | 0.213 | -4.56 | 0.700 | 0.56 | 0.719 |
| History of stroke | Yes vs. No | -34.07 | 0.462 | -16.66 | 0.414 | 0.09 | 0.973 |
| Reduced body height by >3 cm | Yes vs. No | 7.51 | 0.739 | -12.12 | 0.223 | -1.49 | 0.250 |
| Falls | Yes vs. No | 7.82 | 0.782 | -0.43 | 0.972 | 1.75 | 0.283 |
| Family history of osteoporosis | Yes vs. No | 50.86 | 0.207 | -0.11 | 0.995 | 3.21 | 0.165 |
| Family history of fractures | Yes vs. No | -40.85 | 0.398 | -15.93 | 0.454 | -2.29 | 0.409 |

Abbreviation: MET = metabolic equivalent task.

^a^Model covariates included age, sex, body mass index, physical activity, smoking, milk intake, calcium supplement, history of coronary heart disease, history of type 2 diabetes, history of stroke, height loss >3 cm, falls, family history of osteoporosis, and family history of fractures.

**Supplemental Table 5. Associations between fluorescent oxidation products (FlOPs;** **per 1-SD increase in logarithmic scale) and fracture in the unmatched case-control study: Odds ratios (OR) and 95% confidence intervals (CI) from unconditional multivariable logistic regression models***

| **FlOP** | **OR (95%CI)** | ***P*** |
| --- | --- | --- |
| FlOP_320 | 0.60 (0.34, 1.04) | 0.067 |
| FlOP_360 | 1.73 (1.29, 2.30) | <0.001 |
| FlOP_400 | 3.83 (2.52, 5.83) | <0.001 |

Abbreviation: FI = fluorescent intensity. *Model was adjusted for age, sex, body mass index, physical activity, smoking, milk intake, calcium supplement, history of coronary heart disease, history of type 2 diabetes, history of stroke, height loss >3 cm, falls, family history of osteoporosis, and family history of fractures.

**Supplemental Table 6. Association between fluorescent oxidation products (FlOPs; per 1-SD increase in logarithmic scale) and recent low-trauma fracture by fracture site in the unmatched case-control study: Odds ratios (OR) and 95% confidence interval (95%CI) from unconditional multivariable logistic regression models***

| **FlOPs** | **FlOPs in Controls (Fl/ml)**  Median (Interquartile Range) | **OR (95%CI)^*^** | ***P*^*^** |
| --- | --- | --- | --- |
| **Non-hip fracture** (number of cases=21; number of controls= 434) | | | |
| FlOP_320 | 143 (129, 161) | 0.80 (0.43, 1.48) | 0.477 |
| FlOP_360 | 113 (103, 125) | 1.64 (1.16, 2.32) | 0.005 |
| FlOP_400 | 32.9 (30.2, 36.5) | 3.87 (2.24, 6.67) | <0.001 |
| **Hip fracture (**number of cases=23; number of controls= 434**)** | | | |
| FlOP_320 | 143 (129, 161) | 0.39 (0.14, 1.07) | 0.067 |
| FlOP_360 | 113 (103, 125) | 1.83 (1.22, 2.73) | 0.003 |
| FlOP_400 | 32.9 (30.2, 36.5) | 4.64 (2.49, 8.66) | <0.001 |

Abbreviation: FI = fluorescent intensity. *Models were adjusted for age, sex, body mass index, physical activity, smoking, milk intake, calcium supplement, history of coronary heart disease, history of type 2 diabetes, history of stroke, height loss >3 cm, falls, family history of osteoporosis, and family history of fractures.

**
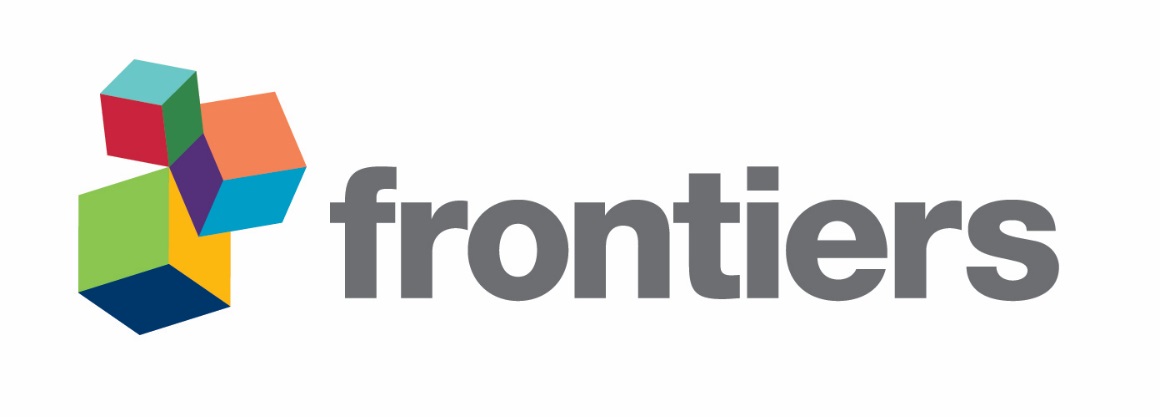
**
